# Interspecies diversity of chloride channel regulators, calcium-activated 3 genes

**DOI:** 10.1371/journal.pone.0191512

**Published:** 2018-01-18

**Authors:** Lars Mundhenk, Nancy A. Erickson, Nikolai Klymiuk, Achim D. Gruber

**Affiliations:** 1 Department of Veterinary Pathology, Faculty of Veterinary Medicine, Freie Universität Berlin, Berlin, Germany; 2 Institute of Molecular Animal Breeding and Biotechnology, Ludwig-Maximilians-Universität, Oberschleissheim, Germany; National Center for Toxicological Research, UNITED STATES

## Abstract

Members of the chloride channel regulators, calcium-activated (CLCA) family, have been implicated in diverse biomedical conditions, including chronic inflammatory airway diseases such as asthma, chronic obstructive pulmonary disease, and cystic fibrosis, the activation of macrophages, and the growth and metastatic spread of tumor cells. Several observations, however, could not be repeated across species boundaries and increasing evidence suggests that select *CLCA* genes are particularly prone to dynamic species-specific evolvements. Here, we systematically characterized structural and expressional differences of the *CLCA3* gene across mammalian species, revealing a spectrum of gene duplications, e.g., in mice and cows, and of gene silencing via diverse chromosomal modifications in pigs and many primates, including humans. In contrast, expression of a canonical CLCA3 protein from a single functional gene seems to be evolutionarily retained in carnivores, rabbits, guinea pigs, and horses. As an accepted asthma model, we chose the cat to establish the tissue and cellular expression pattern of the CLCA3 protein which was primarily found in mucin-producing cells of the respiratory tract and in stratified epithelia of the esophagus. Our results suggest that, among developmental differences in other *CLCA* genes, the *CLCA3* gene possesses a particularly high dynamic evolutionary diversity with pivotal consequences for humans and other primates that seem to lack a CLCA3 protein. Our data also help to explain previous contradictory results on CLCA3 obtained from different species and warrant caution in extrapolating data from animal models in conditions where CLCA3 may be involved.

## Introduction

Members of the family of chloride channel regulators, calcium-activated (CLCA) have been implicated in diverse physiological and pathophysiological cellular functions, including the modulation of transepithelial anion conductance [1], activation of macrophages [2, 3], cell-cell adhesion [4], and cell differentiation [5], including mucus metaplasia [6]. Strong expressional regulations of CLCA proteins have been reported in distinct disease conditions, including chronic airway disease such as asthma and cystic fibrosis (CF) [7] as well as tumor progression and metastasis [4, 5]. Moreover, select CLCA molecules have been suggested as diagnostic markers [5, 8] or even targets in novel therapeutic approaches, particularly in chronic respiratory diseases with mucus overproduction, such as chronic obstructive pulmonary disease (COPD) asthma, and CF [7]. The previous historically-based and partially confusing nomenclature of *CLCA* genes and proteins was recently harmonized to eventually allow for systematic comparisons of orthologs between humans and mice [9]. For example, the originally termed gob-5 mouse protein, later termed mCLCA3, is now designated CLCA1 in the mouse due to its direct orthology to the human CLCA1. Similarly, the previous murine mCLCA1, mCLCA2, and mCLCA4 are now referred to as CLCA3a1, CLCA3a2, and CLCA3b, respectively, in accordance to their orthology to CLCA3 in man. Still, the nomenclature currently used in the databases is complex, in part inconsistent and mostly historically based, especially for duplicated *CLCA3* genes in different species (S1 Table).

One of the most intriguing properties of the CLCA family appears to be its genetic diversity between different mammalian species. First, different numbers of *CLCA* genes have seemingly evolved, for example, with only four *CLCA* genes in man but eight in mice [7]. Second, pseudogene formation and silencing of expression has occurred in some species for members that are fully expressed in other species [10, 11]. Third, contradictory data on alleged functions and regulatory pathways have been found between the human and mouse CLCA1 proteins [12]. Fourth, different cellular expression patterns have been established for the CLCA2 protein between humans, mice, and the pig [13–16] as well as for the CLCA4 protein between humans and mice [17]. We hypothesize that at least some of these differences may result from species-specific evolution of separate *CLCA* genes which would imply restrictions on the translatability of data between different species. In particular, the value of animal models in studying the role of CLCA molecules in disease would be limited and depend on a comprehensive understanding of all relevant differences.

For example, we have recently identified a duplication of the *CLCA*4 gene that appears to be unique to the pig [11] as a model for CF research. In contrast to the human CLCA4 protein which is designated CLCA4a in the pig and expressed at the apical membrane of enterocytes at the villus tips and respiratory epithelial cells [17], the porcine-specific duplication product CLCA4b is selectively expressed in intestinal crypt epithelial cells and has thus adopted a novel cellular expression pattern unlike any other known CLCA protein in other species [18].

Among the four *CLCA* gene clusters in mammals [7, 11], the most striking interspecies differences are evident in the *CLCA3* cluster. In humans and pigs, this cluster contains only a single gene, *CLCA3*, which is thought to be a pseudogene with no functional protein being expressed [10, 11]. In contrast, the murine *Clca3* cluster comprises three closely related genes that appear to be the product of two subsequent duplication events. All three mouse genes, *Clca3a1*, *Clca3a2*, and *Clca3b* are thought to express fully functional proteins [7]. For the CLCA3a1 protein, a strong expression was reported in bronchial and tracheal epithelia as well as in submucosal airway glands and in other tissues with secretory functions, including the mammary gland, intestinal tract, gall bladder, pancreas, and kidney [19]. In addition, germinal centers of lymphatic tissues, spermatids, and keratinocytes of the skin, esophagus, and cornea were found to express the *Clca3a1* transcript [20]. Moreover, the CLCA3a1 protein was also detected in endothelial cells of pleural and subpleural blood vessels and in lymphatic endothelium [21, 22]. Of note, CLCA3a1 and CLCA3a2 share 96% nucleic acid sequence identity on the cDNA level and 92% on the amino acid level [19, 23], raising the question of whether some of the expression data may have resulted from unintended cross-reactivity of the probes used. Not surprisingly, available antibodies failed to discriminate between these two homologs [24]. Only a real–time RT-PCR approach using taqman-probes reliably discriminated between CLCA3a1 which is predominantly expressed in spleen and bone marrow and CLCA3a2 which was found in the lactating and involuting mammary gland [25]. Intestine and trachea expressed similar levels of both homologues [25]. Transcripts of the third murine CLCA3 homolog, CLCA3b, formerly known as mCLCA4, were detected in smooth muscle cells of major blood vessels in the heart, gastrointestinal tract, bronchioles, and in aortic and pulmonary endothelial cells [26]. It therefore seems as if the two gene duplications in the mouse have resulted in three separate murine CLCA3 homologs which are expressed in quite distinct cell types, including epithelial, endothelial, and smooth muscle cells, possibly occupying distinct functional niches that are almost entirely unknown [27]. Clearly, this scenario would widely limit the value of mouse models for studies on CLCA3.

Further unique peculiarities have been identified for the bovine *CLCA3* gene products. CLCA3, originally termed lung-endothelial cell adhesion molecule-1, Lu-ECAM-1, was the first CLCA family member to be discovered on the molecular level [28]. It was cloned in 1997 from bovine aortic endothelial cells and found strongly expressed in endothelial cells of the lung. Functional investigations had identified a role in mediating adhesion of metastatic melanoma cells to lung endothelium [29, 30]. In contrast, its closely related bovine homolog CLCAx, originally termed calcium activated chloride channel, CaCC, with 88% amino acid identity to CLCA3, was isolated from tracheal respiratory epithelium with no expression in the lung [31]. Furthermore, CLCAx had been cloned as putative anion channel with no known link to cell-cell adhesion. Thus, all data available on the two bovine CLCA3 homologs show major discrepancies to their relatives in humans, mice, and pigs, suggesting another unique picture in *bos taurus*.

The origin and evolutionary background of this striking diversity among mammals in the *CLCA3* gene cluster have not yet been addressed and data on *CLCA3* variations in other mammals are lacking. Here, we aimed at a systematic genomic comparison of the *CLCA3* gene cluster among mammals. We hypothesized that a more comprehensive understanding of the evolutionary divergences may help to tackle the widely contradictory data available to date and aid in the interpretation of animal models for relevant human diseases. As we identified the cat as one of the few mammalian species with only a single genuine and apparently fully expressed and functional *CLCA*3 gene, the tissue and cellular expression patterns as well as the predicted protein structure of the feline CLCA3 were established in detail and compared to the scenarios in other mammals, including man.

## Materials and methods

### *In silico* analyses of mammalian *CLCA* genes and proteins

A total of 85 CLCA protein sequences was extracted from the GenBank (www.ncbi.nlm.nih.gov) or ensembl (www.ensembl.org) databases or translated *in silico* from pseudogene sequences. The phylogenetic analyses were performed according to Plog et al. [11] by calculating genetic distance and most parsimony trees upon an alignment which comprise the amino acid sequences of all CLCA variants. Cryptic splice acceptor and donor sites within intron 8 were identified using the human splicing finder (http://www.umd.be/HSF3/) and confirmed with the Berkeley Drosophila Genome Project (http://www.fruitfly.org/seq_tools/splice.html). The structures of the feline CLCA genes were confirmed manually by comparing the entire locus between feline outer dense fiber of sperm tails 2-like, *ODF2L*, and SH3-domain GRB2-like endophilin B1, *SH3GBL1* genes to the genomic organization in the orthologous human locus and to the respective human cDNA sequences. The feline *CLCA* genes were classified as presumably intact if they revealed an open reading frame as well as intact splice sites.

### *In silico* amino acid sequence analyses and generation of antibodies

The amino acid sequences of the feline CLCA3 were analyzed *in silico* using the SignalP 4.1 [32], Kyte–Doolittle [33], SOSUI [34], HMMtop [35], DAS [36], PSORT II [37] and NetNGlyc (http://www.cbs.dtu.dk/services/NetNGlyc) software. Putative proteolytic cleavage sites were identified by interspecies comparison of the amino acid sequences. The location of the protein domains was depicted from the Genbank database (www.ncbi.nlm.nih.gov). For the generation of antibodies against feline CLCA3, two oligopeptides were synthesized based on immunogenicity prediction, named fe3-1, corresponding to amino acids (aa) 224 to 238 (AFIPEKSQTAKDSI) and fe3-2, corresponding to aa 531 to 545 (LLQDPKGTRYETSD). The only immunogenic epitopes which were not found in other CLCA homologs were located in the predicted amino-terminal cleavage product of CLCA3. Oligopeptides were coupled to keyhole limpet hemocyanin (KLH) and used for standard immunization of two rabbits each as described earlier [11]. The immune sera were designated feCLCA3-1a, feCLCA3-1b, feCLCA3-2a, and feCLCA3-2b. The antiserum feCLCA3-2b was affinity immunopurified as described [11]. The resulting immunopurified polyclonal antibodies were named feCLCA3-2b-ap.

### Animals and tissue processing

The following tissues (n = 3 to 5, except for the respiratory bifurcation n = 2) from a total of 11 adult European shorthair cats, *Felis catus*, were shock-frozen on dry ice or immersion-fixed in 4% neutral-buffered formalin: nasal cavity, trachea (dissected into cranial, medial, and caudal parts), respiratory bifurcation, main tracheal bronchus, lung parenchyma, esophagus, stomach, small intestine, caecum, large intestine, skin, mammary gland, heart, femoral skeletal muscle, cerebellum, cerebrum, liver, gall bladder, pancreas, spleen, bone marrow, cornea, ovaries, uterus, heart base aorta, dorsal root ganglion, mandibular glands, parotid salivary gland, mandibular lymph node, thymus, kidney, adrenal gland, urinary bladder, prostate, and vesicular gland. The animals had been autopsied in routine pathological diagnostics or were originally used in a separate study for other purposes (State Office of Health and Social Affairs Berlin, approval number A0274/14). All tissues included here had no histopathological evidence of disease.

All animal studies were conducted in strict accordance with the Federation of European Laboratory Animal Science Associations (FELASA) guidelines and recommendations for the care and use of laboratory animals, and all efforts were made to minimize animal discomfort and suffering. The cats were anesthetized and euthanized by an intravenous overdose of pentobarbital (Narcoren®).

### Tissue expression pattern of feline CLCA3 on mRNA level

Total RNA from tissues listed above was isolated using the NucleoSpin® RNA isolation Kit (Macherey-Nagel, Düren, Germany) according to manufacturer´s instructions. RNA was reversely transcribed as described [3]. Specific primers and probes were designed for feline *CLCA3* and the reference gene *ß-2 microglobulin* (*ß2M*) using Primer3 software (primertool, Whitehead Institute of Biomedical Research; Table 1), encompassing an intron to exclude amplification of contaminating genomic DNA. Quantitative RT-PCR was performed as described [3]. Tissues were defined to express CLCA3 when at least two samples out of 3 to 5 showed cycle threshold (Ct)-values substantially lower than 40 (n = 3 to 5, respiratory bifurcation n = 2).

**Table 1:** Primers, probes, and amplicon sizes of feline *CLCA3* and the reference gene.

Gene	GenBank accession no.	Oligonucleotide	Nucleotide Sequence (5‘-3‘)	Amplicon size, bp
*CLCA3*	XM_003990321	upstream primer	TCCCAGACTGCAAAGGACTC	179 bp
		downstream primer	TGTCATGGGAGGTGCATTCT	
		TaqMan Probe	GCAGAAGCACATGGGATGTA	
*ß2M*	NM_001009876	Upstream primer	CGTTTTGTGGTCTTGGTCCT	115 bp
		downstream primer	TTGGCTTTCCATTCTCTGCT	
		TaqMan Probe	TCAGGTTTACTCCCGTCACC	

### Immunohistochemistry

Formalin-fixed, paraffin-embedded tissues of trachea and esophagus (n = 3 to 5) were cut at 3 μm thickness, mounted on adhesive glass slides, and dewaxed in xylene, followed by rehydration in decreasing ethanol concentrations and blocking of endogenous peroxidase. Each anti-CLCA3 antibody was tested using the following procedural variations: (a) without antigen retrieval, (b) 12 min microwave heating (600 W) in 200 ml 10 mM citric acid, pH 6.0, or (c) 10 min protease treatment at 37°C. Respective pre-immune sera served as controls. Only the feCLCA3-2b antibody showed a specific staining pattern using procedure (a). To further test for specificity, the feCLCA3-2b antibody was pre-absorbed for approximately 2 h with its peptide (50 μg/ml) used for immunization or with an irrelevant peptide. Slides were blocked using Roti-Immunoblock in goat normal serum at a dilution of 1:5 for 30 min at room temperature, followed by incubation with feCLCA3-2b diluted at 1:2,000, with the pre-absorbed antibody or with the pre-immune serum overnight at 4°C. After washing, the slides were incubated with secondary goat anti-rabbit antibodies diluted at 1:200 for 30 min at room temperature. ABC solution (Vectastain Elite ABC Kit, Vector Laboratories Inc., Burlingame, CA) was added for 30 min at room temperature. Diaminobenzidine (DAB) was used for color development. Sections were either counterstained with hematoxylin or treated with the periodic acid-Schiff (PAS) reaction.

### Immunoblot analyses

Tissue samples including cellular and extracellular proteins of trachea, esophagus, and stomach were homogenized in standard lysis buffer, boiled, and immunoblotted as described [11]. Membranes were probed with anti-feCLCA3-2b-ap diluted at 1:1,000, an irrelevant immunopurified antibody diluted 1:1,000 or anti-beta-actin antibody (Sigma-Aldrich, Munich, Germany) diluted to 1:1,000 as loading control in blocking buffer. Membranes were incubated with secondary horseradish peroxidase-conjugated anti-rabbit diluted 1: 2,000 or anti-mouse IgG diluted 1:10,000, respectively, for 1 hour and protein labelling was visualized using enhanced chemiluminescence (Thermo Fisher Scientific, Rockford, IL).

## Results

### High evolutionary dynamics in the mammalian *CLCA*3 gene cluster

**Fig 1:**
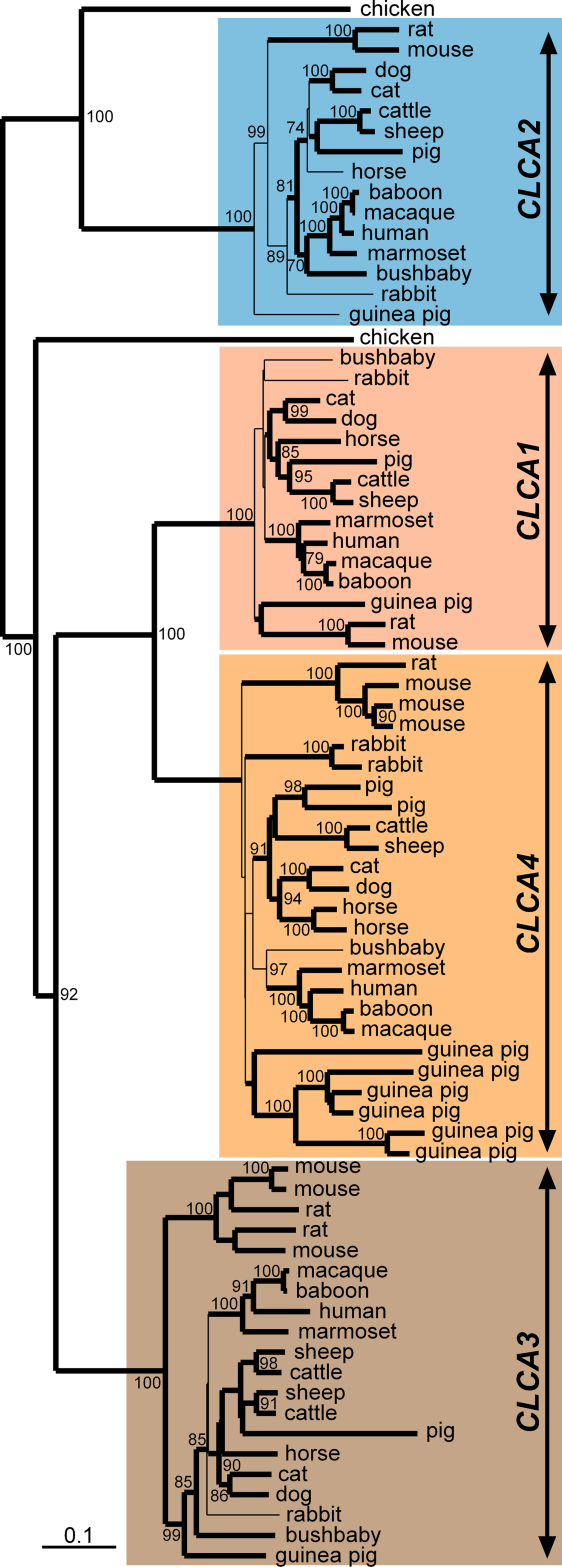
Phylogenetic relationship of mammalian *CLCA* genes. *In silico* analysis revealed four distinct *CLCA* clusters in mammals, named after their human representatives *CLCA1*, *CLCA2*, *CLCA3*, and *CLCA4*. In contrast to mammals, only two *CLCA* genes were identified in chickens. A genetic distance tree was calculated upon the alignment of 77 CLCA protein sequences. Branch nodes that appeared more than 70 times in 100 bootstrapped genetic distance trees are indicated by their bootstrap value and branches that were consistently abundant also in a most-parsimonious tree are shown in bold lines.

**Fig 2:**
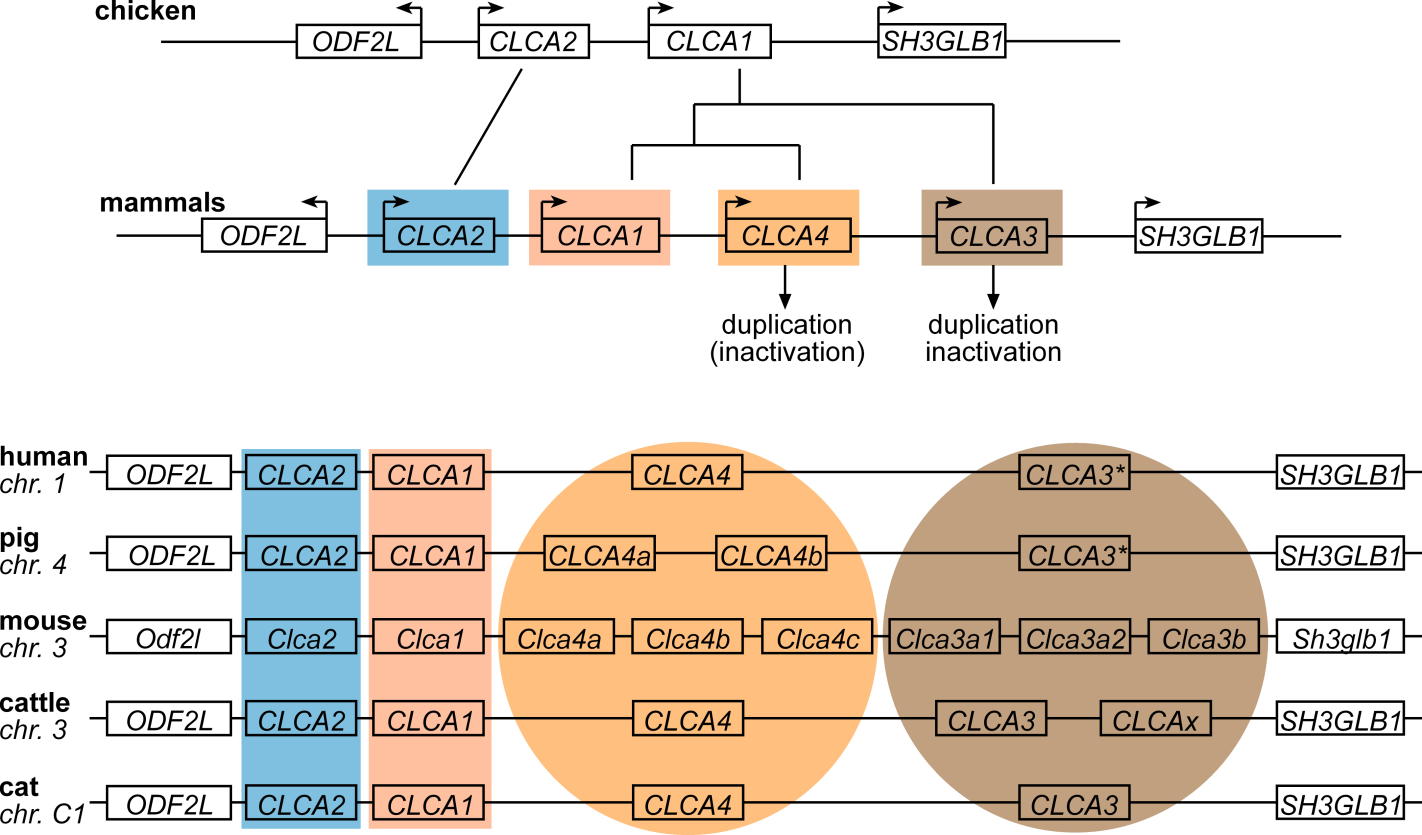
Diverse evolutionary dynamics of *CLCA* genes. In chicken and mammals, all *CLCA* genes are conserved in a single locus, flanked by the *ODF2L* and *SH3GBL1* genes (upper panel). In chickens, only two *CLCA* genes, *CLCA1* and *CLCA2*, are found within this locus with no other *CLCA*-related genes throughout the genome. According to the phylogenetic pattern, *CLCA2* seems highly conserved, with a single intact representative in each mammal examined. In contrast, the ancestor of another vertebrate *CLCA* gene has apparently undergone two independent duplications in early mammalian evolution, resulting in *CLCA1*, *CLCA3*, and *CLCA4*, but it remained as a single copy in chicken. While *CLCA1* is present as a single intact copy in all mammalian species examined and chicken, *CLCA3* and *CLCA4* underwent further duplication or inactivation (*) events in select mammalian branches (lower panel). As the nomenclature of *CLCA* genes is still inconsistent, in particular for duplicated genes, we followed the current designations used in the GenBank database with the exception of bovine unidentified gene *LOC784768* which we here termed *CLCAx* for the ease of understanding (S1 Table).

The *CLCA* gene family can be separated into four distinct clusters in mammals, with designations recently renamed [9] according to the four human representatives, *CLCA1*, *CLCA2*, *CLCA3*, and *CLCA4* (Fig 1). In contrast to mammals, only two *CLCA* genes were identified in chickens (Fig 1) which are organized identically to mammals in a single locus flanked by the genes *ODF2L* and *SH3GBL1* (Fig 2). A single copy of the *CLCA2* gene was consistently found throughout all mammals investigated as well as in chickens, pointing towards its evolutionary conservation (Fig 2). In contrast, the clustering of chicken *CLCA1* with all other mammalian *CLCA* genes suggests that their common ancestor has apparently undergone independent duplications in early mammalian evolution, resulting in the three mammalian genes now termed *CLCA1*, *CLCA3* and *CLCA4* (Fig 2). Similar to *CLCA2*, the *CLCA1* gene also appears to be present as one single intact copy in all mammals investigated here (Fig 2).

**Fig 3:**
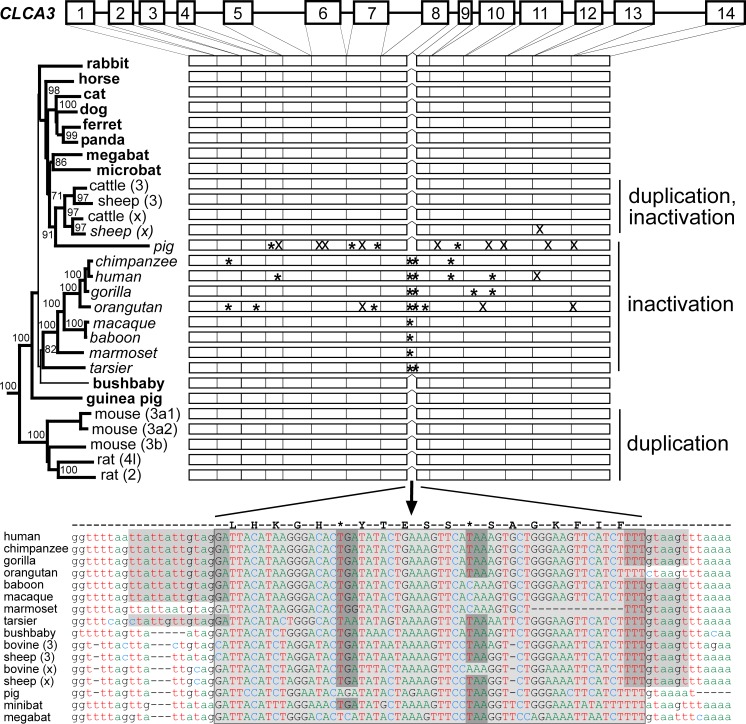
Mechanism of *CLCA3* inactivation in select mammalian species. Duplications of *CLCA3* were found in rodents and ungulates, mutational inactivations in ungulates and in primates (italics), and a single, apparently functional *CLCA3* copy in other mammalian species (bold). The *CLCA3* genes in dry-nosed primates possess an additional exon resulting from cryptic splice sites within intron 8. This genomic segment is also found in other species, including pigs. Each of these species carries at least one stop codon within the predicted exon (shaded within the boxed exon) but cryptic splice acceptor and donor functions are only predicted for dry-nosed primates (gray background). The distinct *CLCA3* gene variants in certain primates displayed further inactivating mutations that were apparently unique for each species. Similar mutational events were found in the porcine *CLCA3* gene and one ovine variant. The nomenclature of the *CLCA3* variants is used according to the GenBank database, except for the bovine unidentified gene *LOC784768* which we termed cattle (x) as well as the ovine unidentified genes *LOC101116002* and *LOC101116267* which we termed sheep (3) and sheep (x), respectively (S1 Table). Box = exons No. 1 to 14 of the *CLCA3* gene, black lines = introns, cross = frame-shift mutations, asterisk = nonsense mutation.

In contrast, marked differences were observed for *CLCA3* and *CLCA4* between distinct mammalian species. Gene duplications had obviously resulted in two or three distinct *CLCA3* gene variants in cows and mice (Fig 2), respectively, and further distinct *CLCA4* gene variants in pigs and mice (Fig 2), supporting previous observations by others [7, 11]. In addition, several kinds of gene inactivation were found in the *CLCA3* cluster in different species (Fig 2). Frame shift mutations and/or one or several premature stop codons are present in the *CLCA3* gene of certain primates, pigs, and one ovine duplicate (Fig 3). *CLCA3* of all dry-nosed primates investigated here, including chimpanzee, human, gorilla, orangutan, macaque, baboon, marmoset, and tarsier, possess an additional exon resulting from an active cryptic splice site within intron 8. This cryptic exon would not change the frame of the *CLCA3* gene but it contains one or two premature stop codons (Fig 3). The *CLCA3* gene of hominids has several additional premature stop codons and frame-shift mutations with rather unique patterns in each species (Fig 3). Among the primate species investigated, the bushbaby, belonging to the suborder of wet-nosed primates, appears to be the only one with an intact and likely functional *CLCA3* gene (Fig 3), as it does not contain an intact splice acceptor site of the cryptic exon. The porcine *CLCA3* also seems to be inactivated by several premature stop codons and frame-shift mutations but, in contrast to primates, the inactivation does not result from the cryptic exon, as there is no evidence of intact splice sites at this exon (Fig 3). As an additional variance, a unique early frame shift mutation was identified in one ovine duplicate (Fig 3). Of note, none of the species with premature stop codons or frame-shift mutations appears to possess a *CLCA3* gene structure that would suggest the expression of a functional protein. Thus, in contrast to carnivores, guinea pigs, rabbits, and horses which all possess a single and seemingly functional *CLCA3* gene (Fig 3), distinct and rather species- specific mechanisms of silencing of the *CLCA3* gene have evolved in most primates, including man, and in pigs and sheep.

### Expression of feline CLCA3 in ciliated epithelial and submucosal cells of airways and keratinocytes of esophagus

We chose the cat to characterize the expression of CLCA3 due to the potential medical relevance of cats as a model for asthma, a condition to which several CLCA members have been linked to in addition to other chronic respiratory diseases including COPD and CF [7]. Feline asthma is a naturally occurring and common condition and can also be induced experimentally, making the cat an accepted animal model that expands the spectrum of models for chronic respiratory disorders [38].

**Fig 4:**
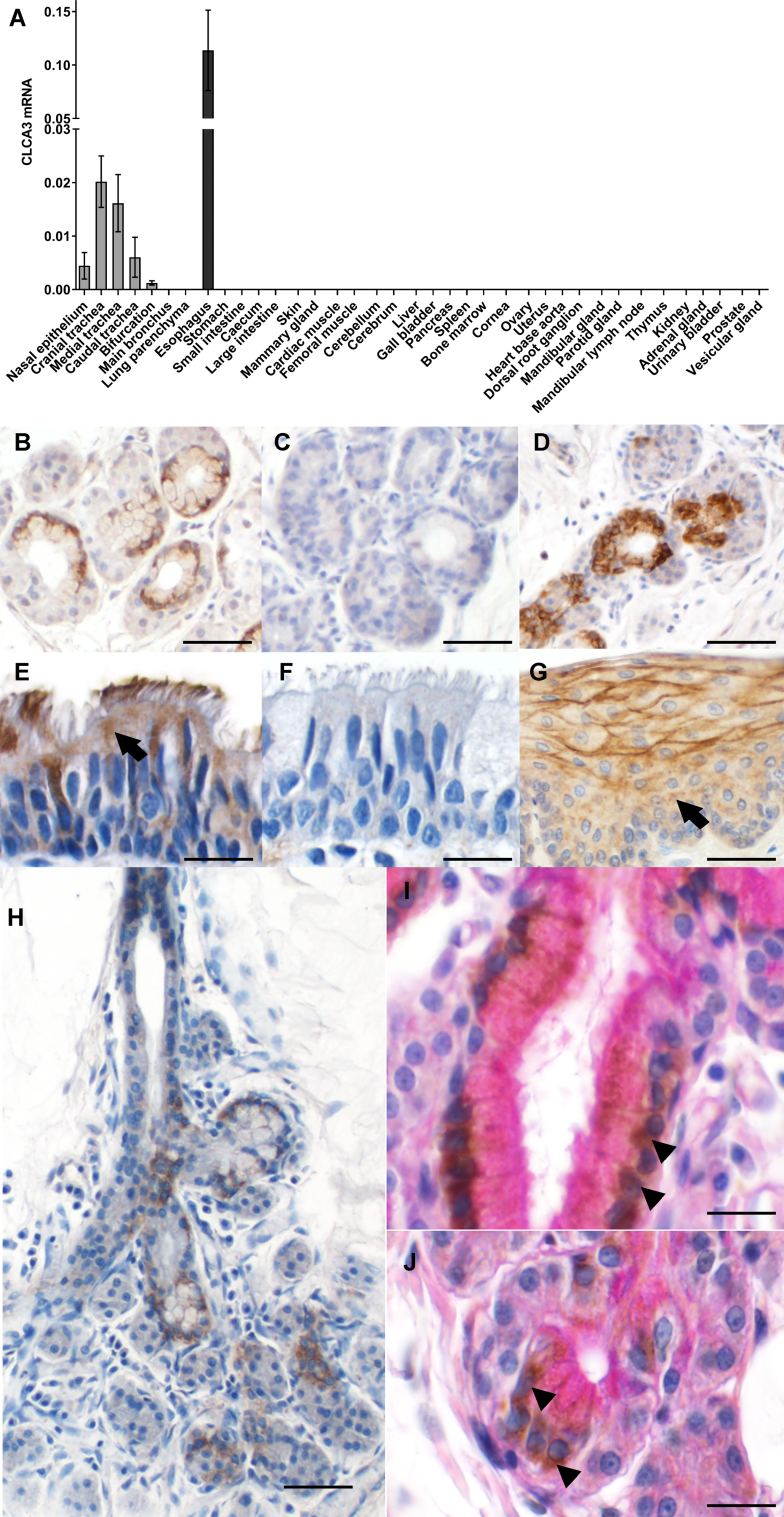
Expression of feline CLCA3 in respiratory epithelial cells and esophageal stratified epithelia. (A) CLCA3 mRNA was detected in the upper respiratory tract. Very high expression levels were found in the esophagus. Data are expressed as mean ± SEM and expression levels are given in ratios of CLCA mRNA copy numbers relative to the copy number of the reference gene ß2M and calculated as 2^Ctß2m-CtCLCA3^ = 2^ΔCt^ (n = 3 to 5, respiratory bifurcation n = 2) (B) Immunohistochemically, the CLCA3 protein was localized to submucosal glands of the respiratory tract. (C) Slides incubated with the pre-absorbed antibodies failed to show the specific staining, whereas (D) preincubation with an irrelevant peptide failed to abolish the specific signal. (E) Single ciliated epithelial cells lining the airways were also positive for CLCA3. The protein was localized in a perinuclear pattern, a vesicular pattern (arrow), or associated with cilia. (F) Sections incubated with the pre-immune serum at the same dilution failed to yield any staining. (G) CLCA3 was also detected in the stratum spinosum of the esophagus where it seemed to be associated with the plasma membrane. A punctate cytoplasmic pattern was observed in deeper cell layers (arrow). (H) The CLCA3 protein was also detected in select cells of submucosal airway glands. PAS reaction counterstain revealed colocalization of CLCA3 (brown color, arrowheads) with (I) mucin-producing tubular and (J) acinar cells (pink color, PAS-positive). Color was developed using DAB as substrate (brown) with hematoxylin (blue, B-H) or PAS (pink, I, J) as counterstain. Bars: B, C, D = 50 μm; E, F = 30 μm; G = 40 μm; H = 60 μm; I, J = 15 μm.

Feline CLCA3 mRNA was detected in the upper respiratory tract from the nasal cavity down to the bronchial bifurcation (Fig 4A) with the following Ct ranges: nasal cavity: 28.48 to 33.21, trachea–cranial part: 28.11 to 31.98,—medial part: 28.52 to 30.89,—caudal part: 29.26 to 35.94, bifurcation: 32.57 to 33.07 but not in main bronchi or lung parenchyma. It was also found to be highly expressed in the esophagus with a Ct range between 26.30 and 31.13. No expression was detected in other tissues, including those in which CLCA3 variants had been reported in other species, such as the mammary gland, spleen, lung, and aorta [10, 25, 26, 28].

To identify the expressing cell types in tissues where CLCA3 mRNA was found, these tissues were analyzed by immunohistochemistry using specifically generated anti-feline CLCA3 antibodies. In the respiratory tract, the CLCA3 protein was localized to cells of submucosal glands throughout the airways and in ciliated epithelial cells (Fig 4B, 4E and 4H). Here, the signals were most prominent adjacent to the nucleus, sometimes with a tendency to a more diffuse cytoplasmic staining. In the esophagus, CLCA3 protein was identified in keratinocytes of stratified epithelia, predominantly located in the stratum spinosum (Fig 4G) where the signals appeared to be membrane-associated. In deeper layers, a more punctate, vesicular expression pattern was identified. A colocalization using the PAS reaction clearly identified CLCA3-positive cells in the respiratory mucosa as mucin-producing cells of the acini and the duct system of submucosal glands (Fig 4I and 4J). In addition the prominent perinuclear staining, a more punctate intracellular pattern was also observed.

**Fig 5:**
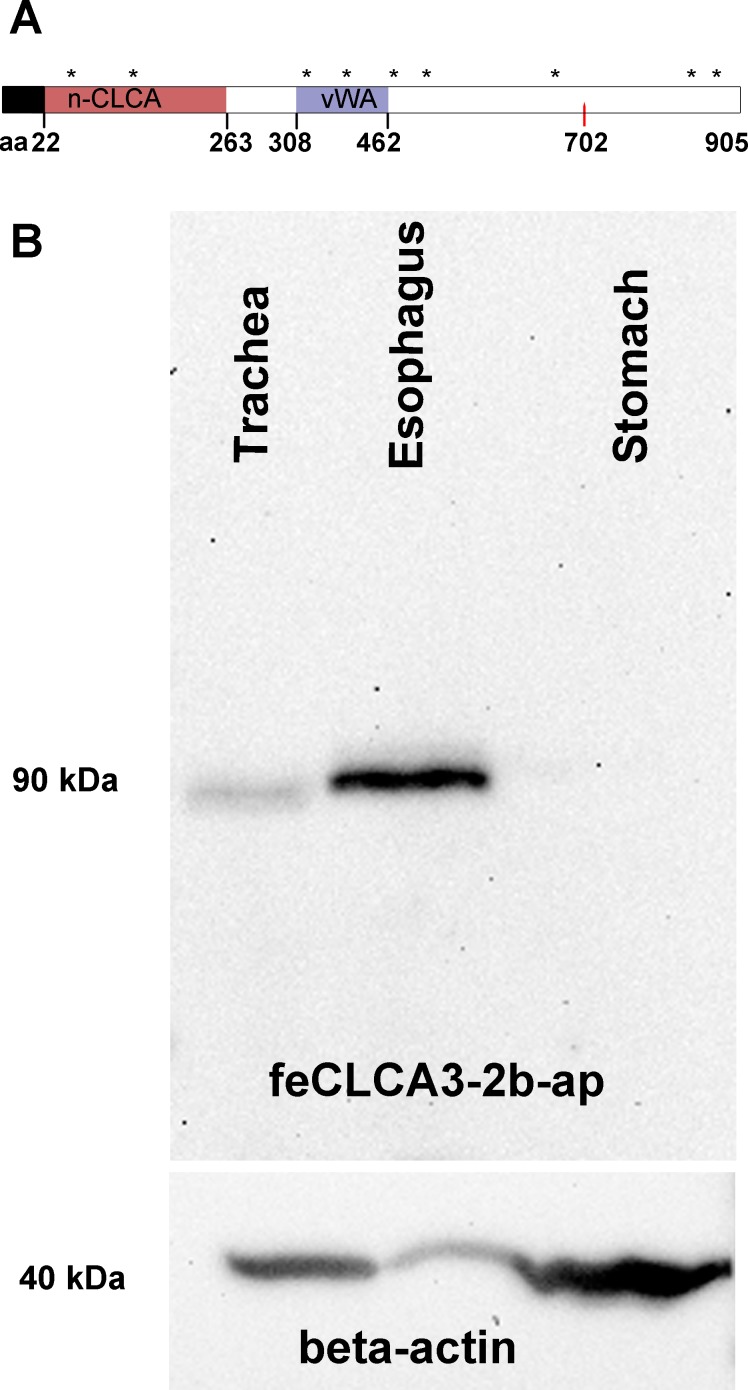
Predicted protein structure of feline CLCA3 and detection of its amino-terminal cleavage product in the respiratory tract and esophagus. (A) The amino acid sequence of feline CLCA3 contains a cleavable signal sequence (black box), an amino-terminal CLCA domain (n-CLCA) followed by a von Willebrand factor type A domain (vWA). No potential transmembrane domain was predicted. Several sites for N-linked glycosylation were predicted (asterisks). The arrow denotes the putative proteolytic cleavage site at 702 aa. aa, amino acids. (B) A 90-kDa protein was detected with the anti-feline CLCA3 antibodies by immunoblotting in the trachea. Much stronger CLCA3 expression was found in the esophagus. The protein was undetectable in the stomach, here shown as tissue without CLCA3 protein expression, as expected from mRNA results. The antibody also detected an approximately 40 kDa band in the tissue lysate of trachea and esophagus, however, these bands were also detected by the irrelevant antibody (S1 Fig). An antibody to beta-actin was used as a loading control (lower panel).

To characterize structural elements of the feline CLCA3 protein, its amino acid sequence was analyzed *in silico*. It starts with a cleavable signal peptide sequence, suggestive of its early translocation into the endoplasmic reticulum (Fig 5A). CLCA3 is predicted to be a fully secreted and soluble protein by the transmembrane prediction softwares used. It contains a hydrophobic region between amino acids 893 and 900 which seems, however, to be too short to represent a transmembrane α-helix that usually contains about 20 amino acids. As reported in the pig and mouse [11, 17, 39], sites of predicted N-linked glycosylation were also found in feline CLCA3 at aa positions 39, 75, 364, 454, 505, 516, 633, 844, and 859 (Fig 5A). Also similar to several previously described CLCA proteins in other species [7, 40], a cysteine-rich domain was found downstream to the signal sequence, designated as n-CLCA (Fig 5A). Adjacent to the n-CLCA domain, a von Willebrand factor type A (vWA) domain was identified (Fig 5A) which is also present in several other CLCA proteins [7]. A unique feature of all known CLCA proteins is their posttranslational cleavage of a 120-kDa precursor protein into a larger, approximately 80-kDa amino terminal and a smaller, approximately 40-kDa carboxy terminal subunit [7]. The exact cleavage site has been experimentally identified only in the murine CLCA1 and the bovine CLCA3 (alias Lu-ECAM-1) [28, 41]. Here, our interspecies protein sequence comparisons revealed a similar site of predicted protein cleavage in the feline CLCA3 protein between amino acids 702 and 703 (Fig 5A). Thus, the feline CLCA3 protein contains several characteristic features that are highly conserved within the CLCA family and thought to be of functional relevance which is still, however, largely elusive [7]. Moreover, it seems to be a soluble protein, possibly secreted similarly to CLCA1 [42] which stands in contrast to CLCA2 and CLCA4 which are thought to be membrane-bound [13, 27].

To characterize the size of the feline CLCA3 protein in tissues, immunoblot analyses were performed on extracts from relevant organs using the immunopurified antibody feCLCA3-2b-ap. An approximately 90 kDa CLCA3 protein was detected in the feline trachea (Fig 5B), consistent with the data obtained by RT-qPCR and immunohistochemistry. The same protein size was found in the esophagus, however, with stronger band intensity (Fig 5B). The size of 90 kDa corresponds well with the predicted size of the amino-glycosylated amino-terminal cleavage product, indicating that this feline variant is posttranslationally cleaved similarly to most other CLCA members investigated so far [7].

## Discussion

We identified a pronounced interspecies diversity of CLCA3 among mammals: some species, including man and pig, appear to live without a CLCA3 protein, whereas others, including the cat, seem to express a single apparently functional protein with several canonical elements of other CLCA proteins. Furthermore, the *CLCA3* gene is duplicated in other mammals, including mice and cows.

### The *CLCA3* Cluster possesses the highest evolutionary dynamics

**Fig 6:**
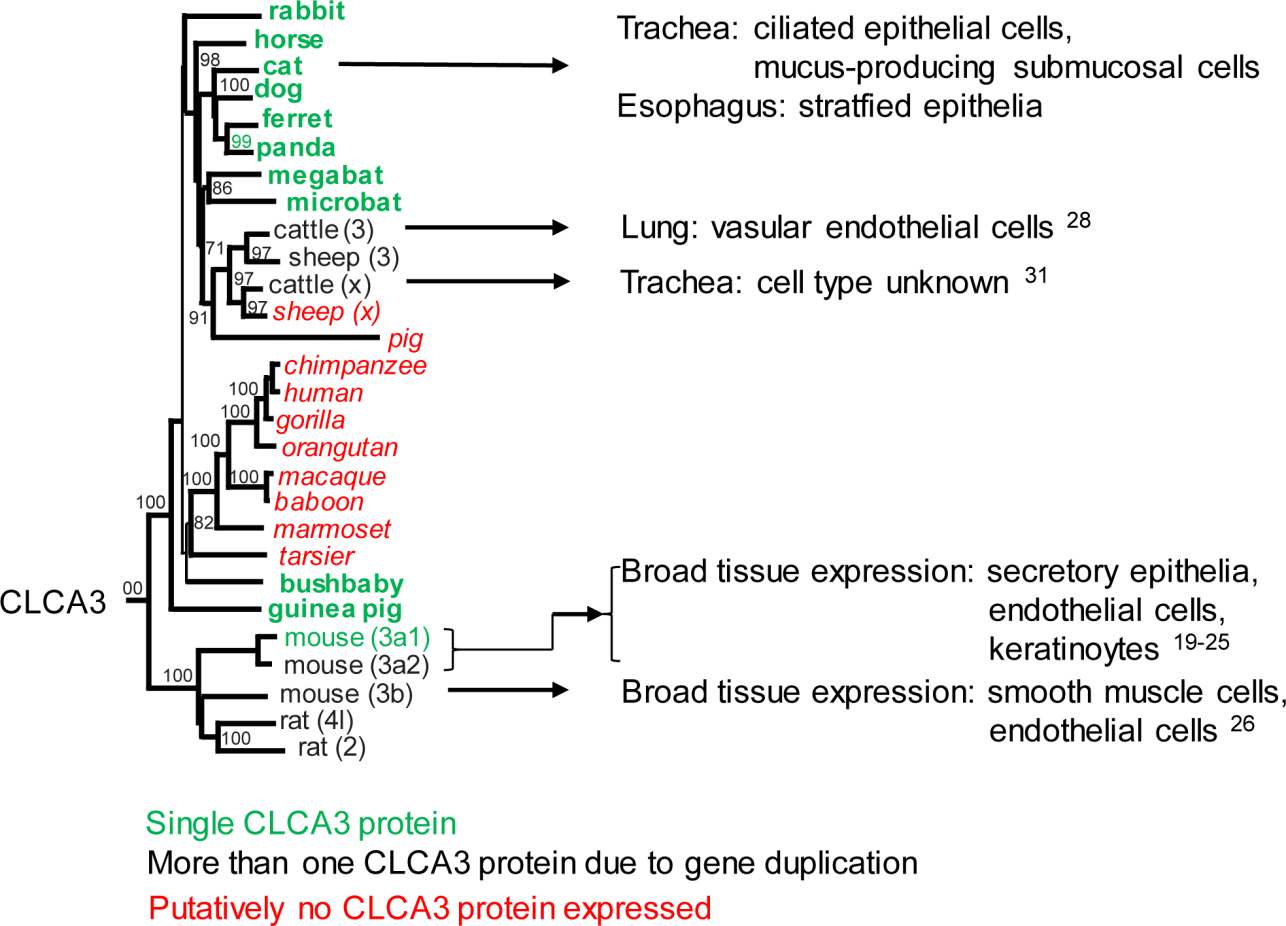
Diversity of CLCA3 protein expression among mammalian species. The CLCA3 protein is expressed as a single version (species colored in green), in duplicated versions (black), or is putatively not expressed at all (red). The duplication events seem to broaden its cellular expression pattern. In the cat, the single CLCA3 protein is found in ciliated epithelial and mucus-producing submucosal cells of the respiratory tract as well as in certain stratified esophageal epithelia. Some orthologs had been detected in the same cell types but further variants had been found in other cell types including endothelial and smooth muscle cells (numbers refer to cited references).

The *CLCA3* cluster seems to possess the highest evolutionary dynamics compared to clusters 1, 2, and 4. This is reflected by 1) the obviously functional expression of a single CLCA3 protein in species such as carnivores, the horse, rabbit, guinea pig, and bush baby, 2) the duplication of the *CLCA3* gene in ruminants and rodents resulting in two or even three apparently functional homologs, and 3) the inactivation of this gene in several species including most primates, pigs, and sheep (Fig 6). In contrast, the *CLCA* cluster 2 seemed to be highly conserved as only a single copy of this gene is present in each of the mammalian species investigated and even in chickens without any evidence of gene duplication or inactivation. Consistently, only a single, apparently functional *CLCA1* gene exists in mammals. Similar to cluster 3, the *CLCA* cluster 4 seems quite diverse with gene duplications in several species, however, mutational genetic inactivation seems to be uncommon.

### Life without a CLCA3 protein

The pseudogene formation of *CLCA3* in humans and pigs had already been described by our group [10, 11]. Here, our extended analysis showed that this formation is not limited to humans in the order of primates. Instead, CLCA3 inactivation was found in almost all primates investigated. Additionally, events of mutational inactivation in ungulates seemed not to be limited to pigs but also occur in sheep.

Inactivation of *CLCA3* in primates seems to be initiated by the insertion of a cryptic exon containing premature stop codons due to an active splice acceptor and donor sites in intron 8. In contrast, the gene was inactivated by an accumulation of premature stop codons and/or frame shift mutations in the pig. This mechanistic difference clearly points towards independent pseudogene formations in primates and ungulates. It will be interesting to explore the evolutionary forces that drove the gene inactivation in these species in contrast to other species, making life without a CLCA3 protein possible.

It still remains questionable whether the *CLCA3* gene has no biological function in primates and pigs at all. It is well established that transcripts of certain pseudogenes may play a role in other cellular mechanisms such as the regulation of gene expression, generation of genetic diversity, or have an impact on gene conversion and recombination [43]. Fragments of *CLCA3* cDNA had previously been amplified from different tissues in humans [10] and further studies need to clarify whether *CLCA3* may be associated with functions other than the formation of a functional protein in primates and pigs.

Interestingly, our *in silico* analyses failed to identify mutations leading to an inactivation of *CLCA3* in the bushbaby. In contrast to all other primates investigated here who are members of dry-nosed primates, the bushbaby belongs to the wet-nosed primates. It thus seems plausible to assume that the pseudogene formation of *CLCA3* had occurred in a common ancestor of dry-nosed but not in wet-nosed primates.

### Expression of a single putatively functional CLCA3 protein in the cat

In addition to the bushbaby, carnivores, horses, rabbits, and guinea pigs were identified as the species with only a single and apparently functional *CLCA3* gene. As an established model of research on chronic airway inflammation [38], the cat was chosen as a candidate for first detailed characterization of the CLCA3 expression pattern and protein structure.

The feline CLCA3 was identified as a protein with no transmembrane domain and several canonical characteristics of other CLCA proteins including an n-CLCA and vWA domain as well as putative posttranslational cleavage [7]. The expression pattern in respiratory ciliated epithelial cells and mucus-producing submucosal gland cells would be in line with proposed cellular functions of CLCA proteins such as activation of airway macrophages or driving mucus metaplasia [2, 3, 12]. Selected CLCA members have also been linked to a functional role in the maturation and differentiation of epithelial cells [5, 13]. The expression of feline CLCA3 in subsets of keratinocytes of esophageal stratified epithelia might point towards such a function. Although our *in silico* analyses predicted CLCA3 to be a soluble protein, an association with the plasma membrane of esophageal keratinocytes and with cilia of respiratory epithelial cells was observed. It remains to be shown whether CLCA3 may interact with integral membrane proteins of these cells. Further studies are clearly needed to elucidate the functions of the CLCA3 protein in the cat and other species in which this protein is evolutionarily retained.

### Species-specific developments and cellular expression patterns of CLCA3

The comparison of the expression pattern of CLCA3 in cats with its orthologs in other species identified some similarities but also marked differences (Fig 6). In humans, no CLCA3 protein expression had been reported, however, cDNA had been detected at low amounts in numerous tissues including the lung, trachea, spleen, thymus, and mammary gland [10]. The murine orthologs CLCA3a1 and 3a2 had been found in tissues with secretory epithelia, including mammary gland, respiratory, and intestinal tract, and other tissues, such as the spleen [20]. On the cellular level, CLCA3a1 had been detected in murine respiratory epithelia of bronchi and trachea as well as in submucosal epithelial cells [19] identical to feline CLCA3 in this study. Additionally, this murine ortholog had been detected in vascular endothelial cells of the lung and in lymphatic endothelial cells [21, 22]. The third murine CLCA3 ortholog, CLCA3b, had been found to be primarily expressed in smooth muscle cells of the cardiac vessels but also gastrointestinal tract, bronchioles, and in aortic and lung endothelial cells [26]. In the cow, the cellular expression pattern is still incompletely known, although the two bovine CLCA3 variants had been one of the first CLCA members to be identified [28, 31]. The bovine CLCAx, previously termed CaCC, was found to be expressed in the trachea but not in the lung [31], whereas CLCA3, the previous LuECAM-1, was clearly expressed in vascular endothelial cells of the lung [28]. The expression of a CLCA variant in mesenchymal cells such as endothelial or smooth muscle cells is unique since CLCA members of other clusters are generally expressed in epithelial cells [7]. The expression pattern of murine and bovine CLCA3 variants in vascular endothelial cells in the lung had originally led to the assumption of them playing a role in the spread of metastatic tumor cells [30], whereas the members found in epithelial cells had been linked to transepithelial anion conductance [31]. It appears likely that genetic duplications of *CLCA3* have broadened the expression to distinct microenvironments with likely even different functions.

In contrast to *CLCA3*, the *CLCA1* cluster is highly conserved with only a single functional member in each species investigated. It is the best investigated cluster in terms of its cellular expression pattern with localization of CLCA1 to goblet and other mucin-producing cells throughout the body in all investigated species so far, including humans [44], pigs [11], mice [45], and horses [46]. Thus, in contrast to the highly conserved *CLCA1* cluster, CLCA3 has undergone a species-specific development with a distinct cellular expression pattern in epithelial cellular niches and, in select species, even in cells of mesenchymal origin (Fig 6).

Of note, the *CLCA4* cluster also showed a high evolutionary dynamic with several events of gene duplication in certain species. The tissue expression pattern showed similarities but also marked differences between the species [17] with duplicates occupying different cellular niches which had been shown for the porcine CLCA4a and 4b [18]. In contrast to *CLCA3*, pseudogene formation seems not to be a common event in the cluster 4 as the murine *CLCA4c* is described to be the only putative pseudogene in the mouse database. Interestingly, our group had identified a naturally occurring deletion mutant of porcine *CLCA4b* among contemporary and ancient pig breeds in high percentages of animals [18]. This protein silencing did not result in any obvious phenotype. Thus, life without pCLCA4b seems possible and it is tempting to speculate that the *CLCA4* cluster will show similar evolutionary dynamics with gene inactivation in the future as described here for *CLCA3*.

### Interspecies diversity–an indicator for possible biological functions?

The tremendous variations of *CLCA3* gene products between species may even add to speculations in terms of their possible functions. For example, a similar interspecies diversity has been established for the bactericidal/permeability-increasing fold-containing (BPIF) protein family, also known as palate, lung and nasal epithelium clone, PLUNC, proteins [47, 48]. Here, 11 genes exist in humans and 14 in the mouse. Both human and mouse gene loci contain lineage-restricted paralogues and pseudogenes [47, 48]. For example, *BPIFA4* had been identified in primates as well as cows and cats, however, it seems to be absent from rodents. Interestingly, in contrast to chimpanzees, gorillas, and rhesus monkeys, a mutational deletion in exon 6 of the human *BPIFA4* gene results in a frame shift with the introduction of a premature stop codon. The human gene does not encode for a functional protein and it had been described as a ‘dying gene’ [47, 48]. Also similar to the CLCA family, *BPIF* genes are encoded in a single, conserved locus in all mammals, including man [47]. Again with strong similarities to several CLCA proteins, BPIF proteins are predominantly expressed in the respiratory passage [49] but may also occur in other tissues such as the thymus [50]. The conspicuous parallels between the BPIF family and the CLCA family in terms of their striking interspecies diversities and their expression in a similar microenvironment, in which innate defense is crucial, may give rise to speculations on related or overlapping functions. Specifically, certain BPIF proteins are thought to play a role in innate host defense and liquid homeostasis in the respiratory tract [51]. The high interspecies diversity and rapid evolution of the BPIF family is comparable and even higher than that of other proteins known to be involved in innate immunity [48], further underscoring speculations on its role in innate host defense. Further work will have to address whether a similar scenario may also be relevant for the CLCA family.

### Consequences for the interpretation of animal models

Members of the CLCA family, especially CLCA1, are known to modulate inflammatory pulmonary diseases such as asthma, COPD, and CF via an as yet unknown mechanism [7]. Mouse and other animal models are widely used to unravel the biomedical significance of these proteins. However, in contrast to humans, certain members of *CLCA* clusters 2 and 4 in the mouse seem to have a redundant function which compensates for the loss of function of CLCA1 [12] and impedes straight interspecies translatability. The tremendous species-specific differences identified here for the *CLCA3* cluster with either no protein expression, expression of a single CLCA3 protein or the expression of several duplicates will certainly have to be considered when interpreting data obtained from animal models. In particular, our data suggest that species-specific variations of CLCA3 may be relevant for comparing different asthma models in animals, including spontaneously asthmatic cats, horses with chronic obstructive bronchitis, and experimental mouse models [38]. In that regard, it will be interesting to explore the functional consequences of the lack of CLCA3 in humans, in contrast to its presence as a single copy gene in cats and horses and multiple duplications in mice.

## Supporting information

S1 Table Nomenclature of duplicated *CLCA3* genes in different species. (PDF)

S1 Fig Original immunoblot of Fig 5. (TIF)
