# Supplementary material for: Interspecies diversity of chloride channel regulators, calcium-activated 3 genes
Source: PLoS One. 2018 Jan 18;13(1):e0191512. doi: 10.1371/journal.pone.0191512 (PMC5773202; doi:10.1371/journal.pone.0191512)
Supplement: S1 Table — (PDF) [file pone.0191512.s001.pdf]

S1 Table: Nomenclature of duplicated *CLCA3* genes in different species

**Species**	**Nomenclature used in this report**	**Nomenclature according to the Genbank database**	**Genbank accession number**	**Previously used names and references**
cattle	*CLCA3* or (*3*)	*CLCA3*	NM_181018	*Lu-ECAM-1* [1] *bCLCA2* [2]
	CLCAx or (*x*)	*LOC784768*	NM_001242583	*CaCC* [3] *bCLCA1* [2]
sheep	*CLCA3* or (*3*)	*LOC101116002*	XM_004002159	-
	CLCAx or (*x*)	*LOC101116267*	XM_012134623	-
mouse	*CLCA3a1* or (*3a1*)	*Clca3a1*	XM_006500968	*mCLCA1* [2] *mCaCC* [4]
	CLCA3a2 or (*3a2*)	*Clca3a2*	XM_006502346	*mCLCA2* [5]
	CLCA3b or (*3b*)	*Clca3b*	NM_139148	*mCLCA4* [6]
rat	*CLCA4l* or (*4l*)	*Clca4l*	NM_001077356	-
	CLCA2 or (*2*)	*Clca2*	NM_001013202	-

1. Elble RC, Widom J, Gruber AD, Abdel-Ghany M, Levine R, Goodwin A, et al. Cloning and characterization of lung-endothelial cell adhesion molecule-1 suggest it is an endothelial chloride channel. J Biol Chem. 1997;272(44):27853-61. PubMed PMID: 9346932.
2. Gruber AD, Fuller CM, Elble RC, Benos DJ, Pauli BU. The CLCA gene family: a novel family of putative chloride channels. Current Genomics. 2000;1(2):201-22.
3. Cunningham SA, Awayda MS, Bubien JK, Ismailov, II, Arrate MP, Berdiev BK, et al. Cloning of an epithelial chloride channel from bovine trachea. J Biol Chem. 1995;270(52):31016-26. PubMed PMID: 8537359.
4. Gandhi R, Elble RC, Gruber AD, Schreur KD, Ji HL, Fuller CM, et al. Molecular and functional characterization of a calcium-sensitive chloride channel from mouse lung. J Biol Chem. 1998;273(48):32096-101. PubMed PMID: 9822685.
5. Lee D, Ha S, Kho Y, Kim J, Cho K, Baik M, et al. Induction of mouse Ca(2+)-sensitive chloride channel 2 gene during involution of mammary gland. Biochem Biophys Res Commun. 1999;264(3):933-7. doi: 10.1006/bbrc.1999.1583. PubMed PMID: 10544033.
6. Elble RC, Ji G, Nehrke K, DeBiasio J, Kingsley PD, Kotlikoff MI, et al. Molecular and functional characterization of a murine calcium-activated chloride channel expressed in smooth muscle. J Biol Chem. 2002;277(21):18586-91. doi: 10.1074/jbc.M200829200. PubMed PMID: 11896056.
