# Supplementary figures and images for: Interspecies diversity of chloride channel regulators, calcium-activated 3 genes
Source: PLoS One. 2018 Jan 18;13(1):e0191512. doi: 10.1371/journal.pone.0191512 (PMC5773202; doi:10.1371/journal.pone.0191512)

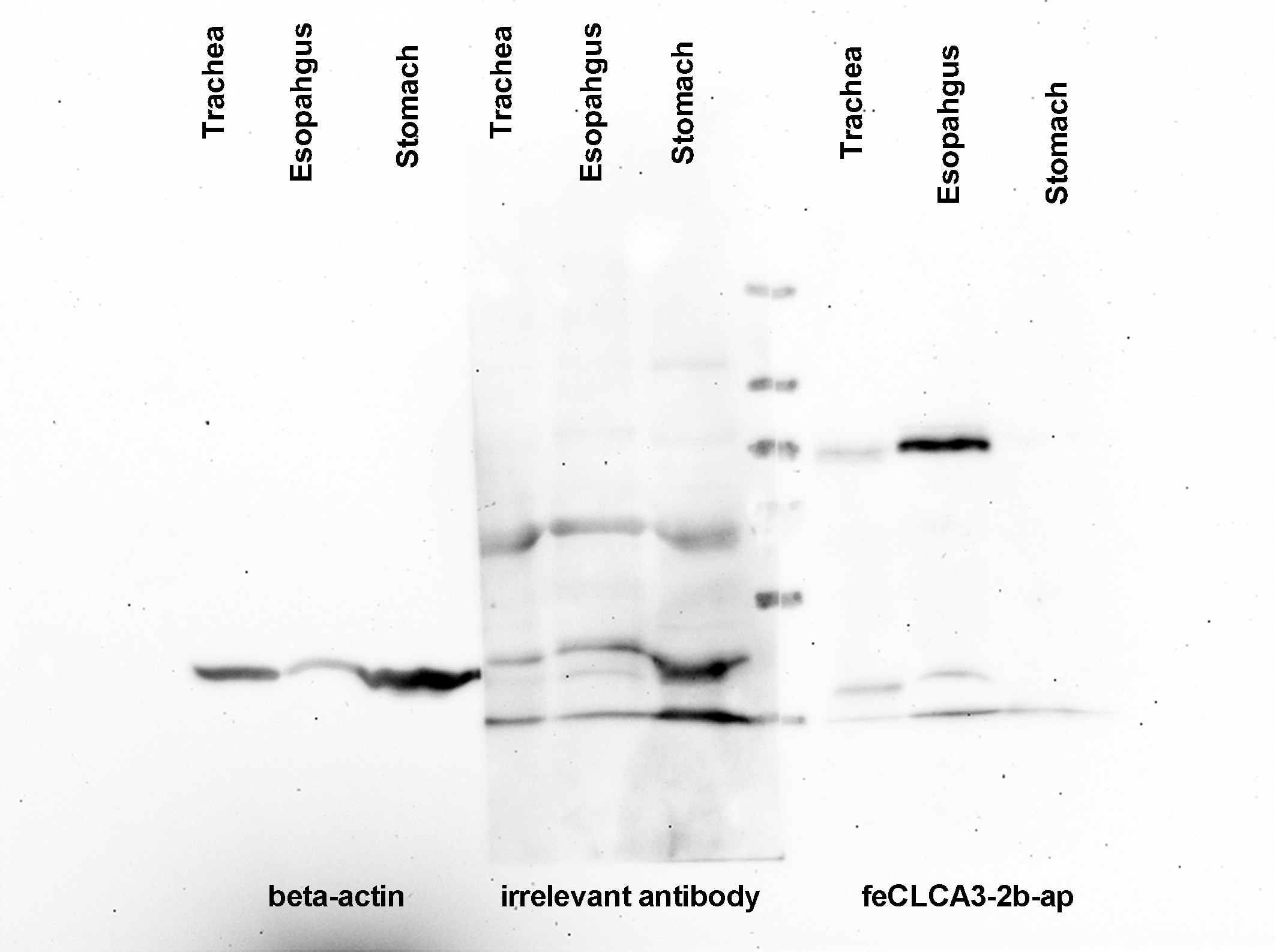

Supplement: S1 Fig — (TIF) [file pone.0191512.s002.tif]
